# Anti-Hyperglycemic Activity of Major Compounds from *Calea ternifolia*

**DOI:** 10.3390/molecules22020289

**Published:** 2017-02-14

**Authors:** Sonia Escandón-Rivera, Araceli Pérez-Vásquez, Andrés Navarrete, Mariana Hernández, Edelmira Linares, Robert Bye, Rachel Mata

**Affiliations:** 1 Facultad de Química, Universidad Nacional Autónoma de México, Mexico City 04510, Mexico; soniaer@ciencias.unam.mx (S.E.-R.); perezva@unam.mx (A.P.-V.); anavarrt@unam.mx (A.N.); anairamyzenaltey@hotmail.com (M.H.); 2 Instituto de Biología, Universidad Nacional Autónoma de México, Mexico City 04510, Mexico; mazari@ib.unam.mx (E.L.); rbyeunam@ib.unam.mx (R.B.)

**Keywords:** *Calea ternifolia*, Asteraceae, chromenes, sesquiterpene lactones, antihyperglycemic

## Abstract

Demethylisoencecalin (**1**) and caleins A (**4**) and C (**5**) (3.16–31.6 mg/kg, p.o.), the major components from an infusion of *Calea ternifolia* controlled postprandial glucose levels during an oral sucrose tolerance test (OSTT, 3 g/kg) in normal and nicotinamide/streptozotocin (NA/STZ, 40/100 mg/kg) hyperglicemic mice. The effects were comparable to those of acarbose (5 mg/kg). During the isolation of **1**, **4**, and **5**, four additional metabolites not previously reported for the plant, were obtained, namely 6-acetyl-5-hydroxy-2-methyl-2-hydroxymethyl-2*H*-chromene (**3**), herniarin (**6**), scoparone (**7**), and 4′,7-dimethylapigenin (**8**). In addition, the structure of calein C (**5**) was confirmed by X-ray analysis. Pharmacological evaluation of the essential oil of the species (31.6–316.2 mg/kg, p.o.) provoked also an important decrement of blood glucose levels during an OSTT. Gas chromatography coupled with mass spectrometry (GC-MS) analysis of the headspace solid phase microextraction (HS-SPME)-adsorbed compounds and active essential oil obtained by hydrodistillation revealed that chromene **1** was the major component (19.92%); sesquiterpenes represented the highest percentage of the essential oil content (55.67%) and included curcumene (7.10%), spathulenol (12.95%) and caryophyllene oxide (13.0%). A suitable High Performance Liquid Chromatography (HPLC) method for quantifying chromenes **1** and 6-hydroxyacetyl-5-hydroxy-2,2-dimethyl-2*H*-chromene (**2**) was developed and validated according to standard protocols.

## 1. Introduction

In Mexico some patients manage their diabetic condition with herbal products or combine their allopathic treatments with them. These patients consider that herb preparations are less toxic, more efficacious and less expensive than allopathic products [1]. Among these herbs is *Calea ternifolia* Kunth, locally known as “prodigiosa”, “zacachichi” and “amula”; the species is perennial and endemic to Mexico.

*Calea ternifolia* has a long history of use in traditional medicine and rituals aimed at dream-based divination [2]. In contemporary Mexico, the infusion prepared from the whole plant, alone or in combination with other herbs, is widely commercialized for treating colics, fever, cough, and diabetes [1,2,3]. Furthermore, a tincture made up from this species is marketed in the US for medicinal purposes, including diabetes treatment. Previous pharmacological investigations showed that the plant exhibited antiinflammatory [4,5], antiplasmodial [6], antileshimanial [7], antimicrobial, antidiarrheal and antinociceptive effects [8]. Recent neuropharmacological evaluation of an aqueous extract of *C. ternifolia* using the mouse models of convulsion, forced swim, elevated plus-maze, muscular strength in a grip and locomotor tests, revealed that the aqueous extract had not significant effects in these assays [9]. However, an organic extract of this herb displayed significant cytotoxicity against human proximal tubule HK-2 cells and increased the level of nephrotoxicity biomarkers [10].

**Figure 1:**
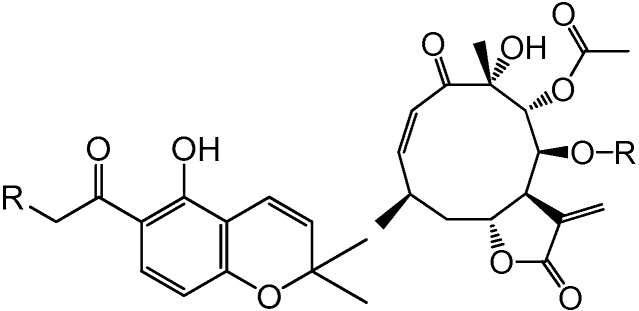
Antihyperglicemic compounds from *C. ternifolia.*

The first written account about the use of *C. ternifolia* for treating diabetes appeared at the end of the last century [3]. In addition, an aqueous extract made up from aerial parts of the plant was effective for controlling fasting and postprandial blood glucose levels in normoglycemic and hyperglycemic mice [11,12]. Bioassay-guided isolation of the active extract using an in vitro enzymatic assay led to the isolation of several flavonoids, chromenes (6-acetyl-5-hydroxy-2,2-dimethyl-2*H*-chromene, demethylencecalin (**1**), 6-hydroxyacetyl-5-hydroxy-2,2-dimethyl-2*H*-chromene (**2**) and 6-acetyl-5-hydroxy-2-hydroxymethyl-2-methyl-2*H*-chromene (**3**)) and sesquiterpene lactones identified as calein A (**4**) and calein C (**5**) (Figure 1) [11,12,13]. Some of these compounds showed potent inhibitory activity against yeast α-glucosidase (α-YG); altogether, these results revealed the antihyperglycemic potential of *C. ternifolia* and its secondary metabolites [11,12]. Other chemical investigations pursued by different groups led to the isolation of several sesquiterpene lactones including caleolactone C and caleins A (**4**) and C (**5**) and analogs; flavonoids; chromenes and chlorogenic acid [7,10,14,15,16,17,18].

Thus, as part of our systematic investigation of Mexican medicinal flora as source of well accepted alternative treatments for type 2 diabetes mellitus and for the discovery of new α-glucosidase inhibitors [13], the goals of this investigation were: (i) to find out if chromene **1**, lactones **4** and **5** and the essential oil of the plant improved postprandial hyperglycemia in vivo after an oral sucrose tolerance test (OSTT); this was in view of the fact that α-glucosidase inhibitor agents improved postprandial hyperglycemia; (ii) to develop quality control pharmacopoeic tests for the crude drug of *C. ternifolia*, considering that the quality of herbs represents the foundation of their efficacy and safety. In this case the quality test consisted in determining the volatile profile of the drug by the analyses of the headspace solid phase microextraction (HS-SPME)-adsorbed compounds and those of the essential oil using gas chromatography coupled with mass spectrometry (GC-MS). On the other hand, for composition an accurate and precise High Performance Liquid Chromatography (HPLC) method for the quantification of compound 1 was proposed; (iii) the final goal was to assess the acute toxicity of an organic extract of the plant considering the significant cytotoxicity recently reported for a methanolic extract of this herb [10]. Altogether these results, will be useful for the rational development of phytopreparations for treating Type-2 diabetes mellitus (T2DM), which nowadays is one of the most important global health problem. Around 415 million people is affected by T2DM worldwide, being Mexico one of the most affected countries. This prevalence is expected to rise beyond 642 million by 2040 [19].

## 2. Results and Discussion

### 2.1. Acute Toxicity Study in Mice

According to the World Health Organization, documentation of a long period of use should be taken into consideration when assessing the safety of herbal drugs. This means that documented experience of long-term use without evidence of safety problems should form the basis of the risk assessment [20]. To our knowledge there are no reports of long-term hazards of *C. ternifolia*. However, Mossoba and coworkers [10] recently reported in vitro toxic effects of a methanol extract prepared from the aerial parts of the plant. In the light of this report, we decided to assess the potential acute toxic effect in mice of an organic extract of the aerial parts of the plant according to the Lorke procedure [21]. The results showed that the treatments did not provoke animal death, behavioral alterations, lesions, or bleeding of the internal tissues and organs of the animals. Thus, the organic extract was not toxic according to the Lorke criteria. These outcomes are consistent with the safe ancestral consumption of this indigenous species and the lack of neuropharmacological effects observed by Salaga and coworkers [9]. It will be appropriated, however, to subject this plant to normal pharmacovigilance practices to provide assurance of its long-term innocuity.

### 2.2. Isolation of Compounds

**Figure 2:**
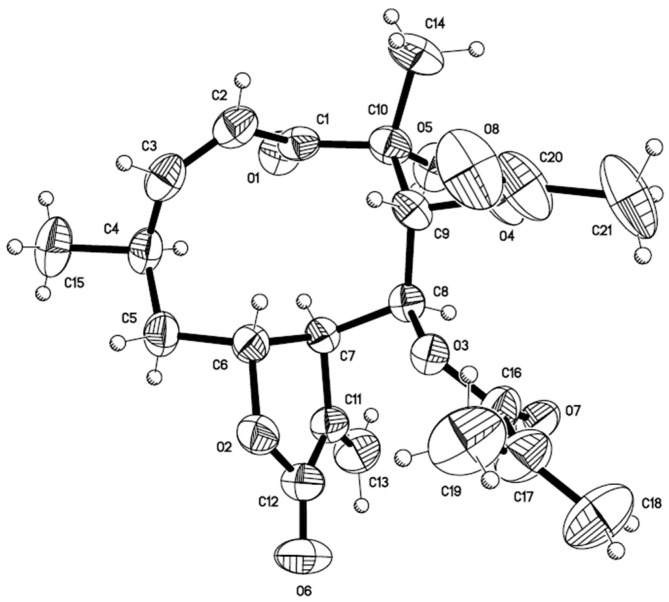
ORTEP drawing of the crystal structure of calein C (**5**).

Next, a chemical analysis of the traditional antidiabetic preparation (infusion) of *C. ternifolia* was undertaken to isolate sufficient amounts of its major components (**1**, **4**, and **5**, Figure 1) for in vivo testing as antihyperglycemic agents. During this process, several minor compounds not previously reported for the plant were isolated. These compounds included 6-acetyl-5-hydroxy-2-methyl-2-hydroxymethyl-2*H*-chromene (**3**) [22,23], herniarin (**6**) [24] scoparone (**7**) [25], and 4′,7-dimethylapigenin (**8**) [26]. In addition, crystals of calein C (**5**) suitable for X-ray analysis were obtained (Figure 2; Tables S1–S5, Supplementary Materials).

### 2.3. Oral Sucrose Tolerance Test of Compounds ***1***, ***4***, ***5*** and Essential Oil

**Figure 3:**
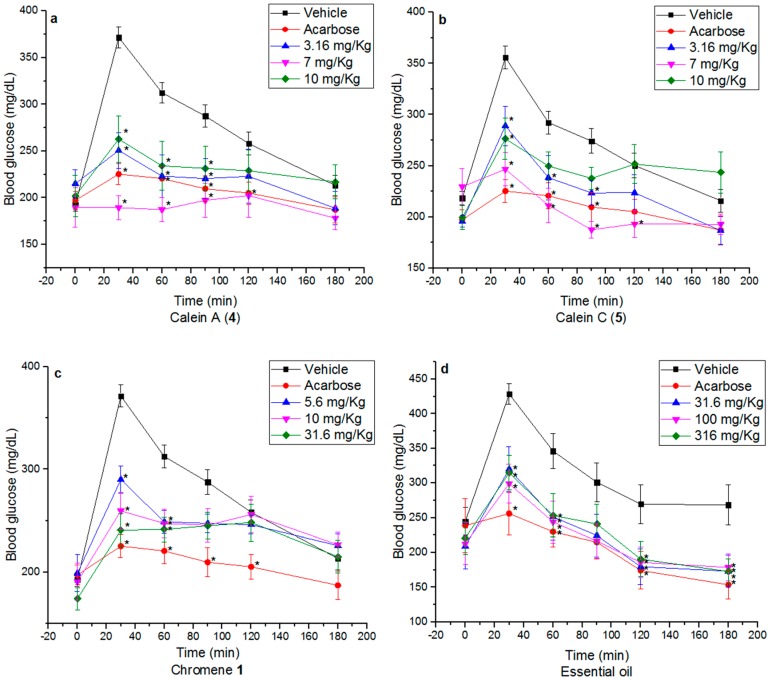
Effect of (**a**) calein A (**4**); (**b**) calein C (**5**); (**c**) chromene **1** and (**d**) essential oil in NA-STZ (40/100 mg/kg) hyperglycemic mice, after a normal sucrose load (3 g/kg). * *p* < 0.05 significantly different ANOVA followed by Dunnett’s t test for comparison with respect to vehicle.

In a previous work, we demonstrated that compounds **1**, **4** and **5** inhibited the activity of α-glucosidase [11,12]. Since α-glucosidase inhibitors are anti-diabetic agents that improve postprandial hyperglycemia, an OSTT was performed in normoglycemic and hyperglycemic (nicotinamide/streptozotocin (NA/STZ), 40/100 mg/kg) mice; these experiments would provide additional evidences of the α-glucosidase inhibitory properties in vivo of these compounds [27]. Caleins A (**4**) and C (**5**) (3.16–10 mg/kg) attenuated the postprandial peak (*p* < 0.05) in both cases (Figure S1, Tables S6 and S7, Supplementary Materials; Figure 3). The effect was more evident in normal mice, which could be due to the metabolic imbalances and pathological changes that occurred in a hyperglycemic state [28,29,30]. Since compounds **4** and **5** exerted similar effects, the nature of the ester moiety at C-8 had no impact in their in vivo action. Likewise, chromene **1** provoked a significant decrease of the postprandial peak at all doses administered (5.6, 10 and 31.6 mg/kg of BW) in both types of animals (Figure 3; Table S8, Supplementary Materials). These results are important since α-glucosidase inhibitors prevent postprandial hypersecretion of insulin and reactive hypoglycemia.

The essential oil (31.6, 100 and 316 mg/kg of BW) also produced an important decrement of the postprandial peak in both normoglycemic (Figure S1, Table S9, Supplementary Materials) and hyperglycemic mice (Figure 3, Table S9, Supplementary Materials) after the sucrose challenge at all doses tested. It is important to point out that the NA-STZ model was selected because it portrays a similar biochemical blood profile and pathogenesis to type 2 diabetes mellitus in humans such as hyperglycemia, glucose intolerance, insulin resistance, dyslipidemia, reduced pancreatic insulin secretion; in addition this model has been validated using anti-type 2 diabetes mellitus drugs [31]. It should be noted that delay absorption of glucose could also involve a change in incretin secretion, an incretin like effect which augments insulin secretion following oral administration of glucose or a stimulation of insulin signaling pathways in peripheral tissues [32]. Further work will be necessary to answer these questions.

### 2.4. Essential Oil Composition

The analysis of the active essential oil via GC-MS analysis revealed that chromene **1** was the major component (19.92%). As summarized in Table 1 and Figure S2 (Supplementary Materials), 31 components were characterized; their relative percentages and retention indexes are indicated in Table 1. Monoterpenes represented 19.97% of the total composition of the essence being camphor (16) the more abundant (12.47%). Sesquiterpenes represented the highest percentage of the essential oil content (55.67%) and included curcumene (24, 7.10%), spathulenol (34, 12.95%) and caryophyllene oxide (35, 13.0%). The yield of the essential oil of *C. ternifolia* was 0.46%. Thus, the high amounts of chromene **1** detected in the oil could account, at least partially, for its pharmacological action. 

### 2.5. HS-SPME Analysis

In order to complete the volatile profile of the plant HS-SPME-adsorbed compounds were analyzed by GC-MS analysis. The divinylbenzene/carboxen/polydimethylsiloxane (DVB/CAR/PDMS) coated fiber extracted the highest total amount of volatile compounds, followed by the polydimethylsiloxane (PDMS). The total amount of volatile compounds extracted with the carboxen/polydimethylsiloxane (CAR/PDMS) and polydimethylsiloxane/divinylbenzene (PDMS/DVB) fibers was lower. Chromene **1** was detected in good yields with fibers PDMS (10.68%) and PDMS/DVB (8.48%), and to a less extend with the DVB/CAR/PDMS (2.68%) one.

### 2.6. Quantification of the Active Markers ***1*** and ***2***

Finally, a comprehensive HPLC method was developed and validated for quantifying chromenes **1** and **2** according to the International Conference on Harmonization guidelines (ICH) [33]. Chromenes **1** and **2** were selected as active markers based on their abundance in the plant and stability [11,12]. Chromene **3** was not included in the validation process as a consequence of its instability. The overall results of the analytical validation are summarized in Table 2 and Table 3 and Figure S3 (Supplementary Materials). The stock solutions and regression equations for each standard compound, together with the limits of detection (LOD) and limits of quantification (LOQ) are shown in Table 2. All calibration curves showed good linearity. All calibration curves showed good linearity within the test ranges (*R*^2^ ≥ 0.9991). The LOD values were 0.3 and 0.1 mg for chromenes **1** and **2** respectively, whereas the LOQ values were 0.8 and 0.3 mg, respectively. Intraday and interday precision relative standard deviations (RSDs) were no more than 0.90% likewise the repeatability variation was no more than 0.90% (Table 2). No significant degradation of **1** and **2** was detected in samples investigated over 24 h at room temperature, and over seven days at −20 °C, compared with the initial values. The method was precise, accurate and linear for the simultaneous quantitative evaluation of the two active markers.

Contents of chromenes in eight different batches of aerial parts from “prodigiosa” were investigated and the results summarized in Table 3; the data indicated that only one of the five samples (IV) purchased in Mexico City markets contained chromenes **1** and **2**, though in lower quantities than wild batches I–III. In batches V–VIII the chromenes were not detected; accordingly, GC-MS analyses of the essential oils of these batches did not showed the presence of **1**, revealing possible substitution of the crude drug. On the other hand, the variation in the chromenes content in batches I–III could be attributed to differences in the recollection date and/or processing of the samples. Thus, the method was successively applied for quantifying **1** and **2** in different batches of the plant.

## 3. Materials and Methods

### 3.1. Chemicals and Reagents

All solvents were purchased from Honeywell Burdick and Jackson (Muskegon, MI, USA). HPLC grade water (18 mΩ) was prepared using an Easypure RF Water Purification System. STZ, ≥98%, NA, ≥98%, acarbose (≥95%), sucrose (ACS reagent), α-bisabolol (12, ≥98%), β-caryophyllene (13, ≥80%), caryophyllene oxide (14, ≥95%), spathulenol (15, ≥96%) and curcumene (16, ≥98%) were purchased from Sigma-Aldrich Chemicals (St. Louis, MO, USA). Formaldehyde solution (37%) was purchased from J.T. Baker (Center Valley, PA, USA). Demethylisoencecalin (**1**) was previously isolated from *C. ternifolia* [12,13]. The purity of the isolated compounds used as markers (**1** and **2**) was determined by HPLC analysis using a normalization method and were calculated to be ≥98%.

### 3.2. General Experimental Procedures

Melting points were determined using a Fisher-Johns apparatus and are uncorrected. Infrared spectra were obtained in the range of 4000 to 400 cm^−1^ using a Spectrum 400 FT-IR instrument (Perkin Elmer San Jose, CA, USA). NMR spectra including NOE differential, COSY, HMBC and HMQC experiments were recorded in CDCl_3_ or CD_3_OD on a Unity Plus 500 spectrometer (Varian, Palo Alto, CA, USA) or on a DMX500 spectrometer (Bruker, Billerica, MA, USA) operating at 500 or 300 MHz (^1^H) or 125 or 75 MHz (^13^C) NMR, using tetramethylsilane as an internal standard. Open column chromatography was carried out on silica gel 60 (70–230 mesh; Merck, Darmstadt, Germany) or Sephadex LH-20 (Sigma-Aldrich Chemical). TLC analyses were performed on silica gel 60 F254 plates (Merck) and spots were visualized by spraying with ceric sulfate (10%) solution in H_2_SO_4_, followed by heating. Semi-preparative RP-HPLC was carried out using a Waters (Milford, MA, USA) HPLC instrument equipped with Waters 996 UV photodiode array detector (900) set at 270–400 nm, a Purospher^®^ Star RP-18 endcapped column (10 mm i.d. × 250 mm, 5 μm) and isocratic conditions (CH_3_CN–H_2_O 48:52; flow: 2.8 mL/min).

### 3.3. Plant Material

Three different batches (I–III) of the aerial parts of *C. ternifolia* were collected in Yecapixtla, Morelos State, Mexico in August 2009, September 2009 and March 2013. In all cases the plant was identified by Drs. R. Bye and E. Linares; voucher specimens (R. Bye and E. Linares 36,059, 36,171 and 2764, respectively) were deposited at the National Herbarium (MEXU), UNAM, Mexico City. In addition, five additional samples of “prodigiosa” were purchased from different markets in Mexico City: Mercado de Sonora (IV and V), Mercado de la Bola (VI) and Pasaje Catedral (VII and VIII).

### 3.4. Preparation of the Extracts and Isolation of the Metabolites

Dried aerial parts (50 g) of *C. ternifolia* were macerated twice during 4 days with 500 mL of CH_2_Cl_2_–MeOH (1:1). After filtration and elimination of the solvent 3.5 g of an organic extract were obtained. On the other hand, dried aerial parts (270 g) of *C. ternifolia* were extracted with 7 L of boiling water (100 °C) during 30 min; after filtering and partitioning with CH_2_Cl_2_ (3 × 7 L) a CH_2_Cl_2_-soluble fraction (DSF) was obtained. From the infusion spontaneously crystallized 4.5 mg of **9**, and during the partitioning process 5 mg of **10** precipitated. DSF was dried over anhydrous Na_2_SO_4_ and concentrated in vacuo to yield 2.22 g. DSF (2 g) was submitted to a column chromatography on silica gel (120 g, eluting with Hexane–CH_2_Cl_2_ (90:10→0:100)) to yield eleven fractions (F1–F11). From fraction F2, further amount of **1** (43 mg) crystallized with *n*-hexane. From the remaining part of this fraction 20 mg of 2 were crystallized. Fraction F4 (225.7 mg) was subjected to silica gel CC by elution with *n*-hexane–CH_2_Cl_2_ (100:0→0:100) to obtain seven tertiary fractions (F4-I–F4-VII). Fraction F4-III gave 3.1 mg of **8** (scoparone); fraction F4-IV yielded 11.1 mg of **3**; finally, F4-V yielded 10 mg of **7**. Fraction F5 was a mixture of sesquiterpene lactones **4**–**6**. This mixture was separated by HPLC (CH_3_CN–H_2_O 48:52, 2.8 mL/min) to give **4** (136 mg; *R*_t_: 10.55 min), **5** (247 mg; *R*_t_: 11.90 min) and **6** (8 mg; *R*_t_: 13.58 min).

### 3.5. Preparation of the Chromene-Rich Fraction

In order to prepare a chromene-rich fraction (CRF), 8 g of dry plant material were extracted with 0.2 L of water as indicated above but the partition process was performed with hexane (0.2 L × 3); the resulting organic fraction was dried over anhydrous Na_2_SO_4_ and concentrated in vacuo. The same process was employed for the preparation of CFFs from all batches. The yields (% CRF) are reported in mg of CRF/g of plant material (Table 3).

### 3.6. X-ray Crystallographic Data of Calein C (***5***)

Single crystal X-ray data were taken with a Bruker (Billerica, MA, USA) Smart Apex CCD diffractometer 01-670-01, using a refinement method of full-matrix least-squares on F2. The compound crystallizes in the orthorhombic system, space group P212121, with a = 10.1880(3) Å a = 90° b = 12.3924(4) Å b = 90° c = 17.8199(6) Å g = 90°, for Z = 4 and F.W. = 406.42. T = 298 K. The calculated density was 1.200 mg/m^3^. The size of crystal used for collection was approximately 0.316 × 0.216 × 0.212 mm. Of the 22,646 collected reflections, 5585 were unique (R_int._ = 0.0524). The absorption coefficient was 0.092 mm^−1^. The structure was solved by direct methods and expanded using Fourier techniques. In the supplementary material are provided crystal data and structure refinement (Table S1), Atomic coordinates (×10^4^) and equivalent isotropic displacement parameters (Å^2^ × 10^3^) (Table S2), Bond lengths [Å] and angles [°] (Table S3), Hydrogen coordinates (×10^4^) and isotropic displacement parameters (Å^2^ × 10^3^) (Table S4), Hydrogen bonds for calein C (**5**) (Table S5). CCDC 1435012 contains the supplementary crystallographic data for **5** in this paper. These data can be obtained free of charge via http://www.ccdc.cam.ac.uk/conts/retrieving.html (or from the CCDC, 12 Union Road, Cambridge CB2 1EZ, UK; Fax: +44 1223 336033; E-mail: deposit@ccdc.cam.ac.uk).

### 3.7. Volatile Composition

#### 3.7.1. Essential Oil

The essential oil was prepared using 50 g of dried aerial parts, which were placed into a 1 L round bottomed flask with 500 mL of distilled water (H_2_O_d_) and then hydrodistilled during 2 h. The oil was separated by liquid-liquid partitioning with CH_2_Cl_2_, which was dried over anhydrous Na_2_SO_4_ and concentrated in vacuo to yield 0.2235 g (0.45% of the dry wt. of the plant) of essential oil.

#### 3.7.2. Headspace Solid-Phase Microextraction

Four fibers were evaluated in this research: polydimethylsiloxane (PDMS, 100 μm) carboxen/polydimethylsiloxane (CAR/PDMS, 75 μm), polydimethylsiloxane/divinylbenzene (PDMS/DVB, 65 μm), and divinylbenzene/carboxen/polydimethylsiloxane (DVB/CAR/PDMS 50/30 μm) (Supelco, Bellefonte, PA, USA). All fibers were conditioned in the GC injector at the temperature and time recommended by the manufacturer. In each case, 40 mg of dried plant material, 100 mg of sodium chloride, and 5 mL of distilled water were transferred to a 10 mL vial, and sealed hermetically with a polypropylene hole-cap and PTFE/coated silicone septa; then, the fiber was exposed to sample headspace for 15 min at 45 °C in a thermostatic bath. After the sampling period, the SPME fiber was inserted into the GC injector port and maintained during 2 min at 250 °C for desorption of compounds.

#### 3.7.3. Gas Chromatography-Mass Spectrometry Analysis

The analyses of oil and volatile compounds were performed on an Agilent (Santa Clara, CA, USA) 6890 N gas chromatograph (GC) with an automatic liquid sampler Agilent 7683B coupled to a LECO Pegasus 4D mass spectrometer. The separation was achieved on a DB-5 capillary column (30 m × 0.25 mm i.d., 0.25 μm film thickness). Gas chromatograph oven initial temperature was kept at 40 °C for 3 min, then increased to 300 °C at 20 °C/min and held for 5 min. The injector temperature was maintained at 300 °C, using splitless injection mode (2 min). Helium was used as carrier gas with a constant flow-rate of 1 mL/min. The mass spectrometer was operated in scan mode from 45–500 *m/z*; ion source temperature was set at 200 °C; the ionization was performed in the impact ionization mode (EI) with the ionization voltage set to 70 eV.

### 3.8. HPLC Analysis

High performance liquid chromatography (HPLC) was performed using a Waters (Milford, MA, USA) 600 HPLC instrument equipped with a Waters 2487 UV diode array detector (DAD). Separation was carried out using a Symmetry C8 (3.9 mm i.d. × 150 mm, 5 μm) column. Elution was carried out at a flow rate of 0.7 mL/min with acetonitrile as solvent A and water as solvent B containing 0.3% phosphoric acid (*v/v*) and using a gradient elution of 45% A at 0–7 min, 45%–50% A at 7–11 min, 50%–80% A at 11–14 min, 80%–45% A at 14–19 min, 45% A at 19–21 min and 45% A at 21–35 min. Each run was followed by re-equilibration period of 15 min. The detection wavelength was set at 265 nm. Injection volume was 20 μL. System control, data collection and data processing were accomplished using Waters Empower 2 chromatography software (Milford, MA, USA).

### 3.9. HPLC Method Validation

The method was validated according to ICH guideline for selectivity, linearity, accuracy, precision, LOD and LOQ [33]. Selectivity was checked using the CRF fraction and a mixture of standards **1** and **2**. Linearity of the method was performed by analyzing a standard solution of the markers in a concentration range of 20–65 μg/mL for standard 1 and 10–80 μg/mL for standard **2**. A calibration line was made and the least square line and correlation coefficient were calculated. The intercept and the regression coefficient were established using the Student’s *t*-test. Both the calibration line and the residuals were graphically inspected and evaluated. The Accuracy was evaluated by means of recovery assays carried out by adding known amounts of the standards of **1** and **2** to the sample at three different levels of the initial concentration of the sample (20%, 40% and 60%). Average recoveries were calculated by the formula: recovery (%) = {(amount found—original amount)/amount spiked} × 100. Precision was evaluated using repeatability (intra-day) and intermediate precision (inter-day). Intra-day and inter-day variations were established using six replicates within the same day and three consecutive days (a total of 18 determinations), respectively. The stability was tested by analyzing the sample solution at different time points (0, 24, 48 and 72 h) and peak areas of all standards were recorded and compared. The % RSD ≤ 2.0 was taken as a measure of precision and stability. LOD and quantification LOQ were determined based on the standard deviation (σ) of the response and the slope (S) (LOD = 3.3 × σ/S and LOQ = 10 × σ/S, respectively).

### 3.10. In Vivo Assays

#### 3.10.1. Experimental Animals

ICR male mice, weighting between 20 and 25 g, were purchased from Centro UNAM-Envigo (Envigo RMS, Indianapolis, IN, USA) and kept in an environmentally controlled room maintained at 22 ± 1 °C with alternating 12 h light/dark natural cycle, with free access to standard rodent pellet diet (Teklad 2018S, Envigo) and water ad libitum. All studies were conducted according to the principles and guidelines of the Mexican Official Norm for Animal Care and Handling (NOM-062-ZOO-1999) [34] with the approval of the Institutional Ethical Committee for the Use of Animals in Pharmacological Testing, Facultad de Química, UNAM (FQ/CICUAL/132/16 approved on March 8, 2016). The assays were conducted in normoglycemic and hyperglycemic mice [35]. 

#### 3.10.2. Nicotinamide-Streptozotocin (NA-STZ) Experimental Induced Hyperglicemia in Mice

Hyperglycemia was induced by i.p. administration of STZ (100 mg/kg) dissolved in 0.1 M citrate buffer, pH 4.5, and maintained on ice prior to use, 15 min after an i.p. administration of NA (40 mg/kg) dissolved in distilled water [36]. One week later, the blood glucose levels in each mouse were measured by the enzymatic glucose oxidase method using a commercial glucometer. Blood samples were collected from the tail vein by means of a small incision at the end of the tail. Only, those mice with blood glucose levels higher than 140 mg/dL were included in the study [35].

#### 3.10.3. Oral Sucrose Tolerance Test

The oil and isolated compounds **1**, **4** and **5** were suspended in saline solution. Acarbose (5 mg/kg) was used as an antihyperglycemic drug. Sucrose (3 g/kg) was used for the carbohydrate tolerance tests. Control mice group received only saline solution. The administration was orally in all cases [35].

Normal and NA-STZ hyperglycemic mice were placed in acrylic boxes forming groups of six animals (I–VI). Group I was administrated with the vehicle; group II received acarbose (5 mg/kg); group III–V received different amounts of oil and compounds (31.6, 100 and 316 mg/kg of BW for oil; 3.16, 7 and 10 mg/kg of BW for **4** and **5**; 5.6, 10 and 31.6 mg/Kg of BW for **1**). The dosage scheme was chosen according to a standard protocol of allometric scaling [37]. Time 0 min was set before treatment with the compounds or control; 30 min later a sucrose load (3.0 g/kg) was given to the animals. Blood samples were obtained 30, 60, 90, 120, and 180 min after the carbohydrate load. The variation of glycemia was calculated with respect to the initial (0 h) glucose level, according to the following equation:

Variation of glycemia = (G*_t_*/G*_i_*)

where G*_i_* is the initial glycemia value and G*_t_* is the glycemia value after treatment.

#### 3.10.4. Statistical Analysis

Results are expressed as the mean ± SEM of six animals in each group. Analysis of variance (ANOVA, one way) was used to analyze changes in blood glucose level followed by Dunnett’s test, *p* < 0.05 was considered statistically significant. SigmaStat software (AnalystSoft Inc., Walnut, Canada) was used for the date analysis.

#### 3.10.5. Acute Toxicity Assay

Mice were divided into control and test groups (*n* = 3) and treated in two phases according to the Lorke protocol. In the first one, the control group and the test group received intragastric doses of vehicle, 10, 100 and 1000 mg/kg of an aqueous extract of *C. ternifolia*. In the second, the animals received doses of 1600, 2900 and 5000 mg/kg of the same treatments. In each phase, mice were observed daily during 14 days for mortality, toxic effects and/or changes in behavioral pattern. At the end of the each phase, the animals were sacrificed in a CO_2_ chamber and the main organs (liver, heart, lung, spleen and kidneys) were observed macroscopically and compared versus those of the control group [20].

## 4. Conclusions

Chromenes **1** and **2** as well as caleins A (**4**) and C (**5**) are the major constituents in the traditional preparation of *C. ternifolia*. It was demonstrated for the first time that these compounds and the essential oil of the plant reduced postprandial hyperglycemia, one of the most common abnormalities in the early phase of type 2 diabetes, after a sucrose challenge, These results are in agreement with the previously demonstrated in vitro α-glucosidase inhibitory activity of **1**, **2** and **5** [11,12], although other anti-hyperglycemic mode of action could not be ruled out. A fast and reliable HPLC method for quantifying the contents of the major active chromenes in aqueous extracts from the aerial parts of *C. ternifolia* was developed and validated. This method was successfully applied to determine the amounts of **1** and **2** in commercial crude drug samples of *C. ternifolia*. The volatile composition of the plant was assessed and the chromene **1** was found to be the more relevant constituent. The latter information would be useful for the development of pharmacopeic and World Health Organization monographs. Thus, this investigation provides relevant information regarding the efficacy, safety and quality control procedures of *C. ternifolia*, which will also contribute to the rational use of the plant.

## Figures and Tables

**Table:** 

	**R**			**R**
**1**	H		**4**	CH_2_=C(CH_3_)CO
**2**	OH		**5**	CH_3_CH=C(CH_3_)CO

**Table 1:** Volatile components identified in *C. ternifolia* by HS-SPME-GC-MS.

No.	Compounds	IR	DVB/CAR/PDMS	PDMS	CAR/PDMS	PDMS/DVB
12	α-Pinene	996	4.25	0.44	3.55	3.19
13	Camphene	1018	4.35	0.45	2.56	1.60
14	β-Pinene	1046	7.19	0.82	2.41	1.21
42	*o*-Cymene	1109	0.95	-	-	5.45
43	*d*-Limonene	1111	6.80	-	-	5.45
15	Eucalyptol	1113	1.91	0.36	2.00	5.45
16	Camphor	1242	4.06	1.79	9.89	5.10
17	Pinocarvone	1261	1.40	-	1.84	0.88
18	1,2,3-Trimethylcyclopentane	1277	-	0.31	2.75	1.22
19	*S*-Pinocamphone	1278	1.16	0.29	2.75	-
44	4-Carvomenthol	1288	0.32		-	-
45	Thymol	1429	0.25	-	-	-
46	α-Cubebene	1526	-	3.83	-	-
20	β-Caryophyllene	1527	3.31	3.83	4.01	1.99
21	β-Cubebene	1539	1.17	1.24	-	-
22	α-Ocimene	1563	0.98		4.07	1.79
23	Germacrene D	1580	1.33	0.41	4.07	1.55
24	Curcumene	1591	5.02	10.90	1.04	8.90
47	α-Zingiberene	1604	1.11	1.80	-	-
25	α-Selinene	1607	0.66	0.79	1.19	-
26	Germacrene A	1627	0.67	1.25	0.42	-
27	δ-Cadinene	1632	1.79	-	2.71	2.61
28	Calamenene	1639	0.33	0.81	1.42	0.88
29	*cis*-α-Farnesene	1641	1.37	1.83	-	0.50
30	α-Calacorene	1666	0.11	0.01	-	0.08
31	Hedicariol	1678	1.36	1.22	2.18	-
32	(*E*)-Nerolidol	1682	2.68	1.97	1.61	1.94
33	Aromadendrene oxide 2	1698	0.22	0.13	-	-
34	Spathulenol	1714	4.40	7.58	3.83	4.12
35	Caryophyllene oxide	1716	3.77	7.69	3.54	5.02
48	*trans*-Chrysanthemal	1723	-	7.64	14.35	1.89
49	Carotol	1738	3.15	4.38	4.00	7.00
50	Humulene epoxide II	1748	0.87	0.93	-	-
51	α-Muurolol	1780	-	2.73	-	-
38	τ-Cadinol	1783	2.90	-	-	3.31
39	β-Eudesmol	1800	1.12	3.81	2.14	5.49
40	α-Bisabolol	1829	0.52	-	-	-
41	Ledene oxide II	1843	0.56	-	-	-
1	Demethylisoencecalin	1883	2.68	10.68	-	8.48

**Table 2:** Validation report of the method for simultaneous determination of chromenes **1** and **2**.

	*R*_t_	Linear Range (mg/mL)	Calibration Equation	*R*^2 a^	LOD (mg/mL)	LOQ (mg/mL)	Precision		Recovery (%mean)	Stability (%RSD)
							Intraday (%RSD)	Interday (%RSD)		
**1**	18.8	20–65	*y* = 206712*x* − 640270	0.9991	0.3	0.8	0.7	0.6	100.20	0.91
**2**	7.0	10–80	*y* = 190874*x* − 138782	0.9997	0.1	0.3	0.9	0.9	100.03	0.20

^a^ *R^2^* correlation coefficient for five data points in the calibration curves (*n* = 3).

**Table 3:** Content of chromenes **1** and **2** in eight batches of “Prodigiosa”.

Batch	% CRF ^a^	Content in mg/mg ^b^	
		1	2
I	0.04	21.91 ± 1.07	22.74 ± 1.62
II	0.17	7.74 ± 0.89	28.99 ± 1.23
III	0.13	58.04 ± 1.62	40.90 ± 1.10
IV	0.14	8.46 ± 1.48	5.04 ± 1.28
V	0.33	nd	nd
VI	0.38	nd	nd
VII	0.32	nd	nd
VIII	0.24	nd	nd

^a^ Yields in mg of chromene-rich fraction/g of plant material; ^b^ (mg/mg of CRF); Data are mean ± SD; *n* = 3; nd = no detected.

## Supplementary Materials

Supplementary materials are available online.
