# Supplementary material for: Anti-Hyperglycemic Activity of Major Compounds from Calea ternifolia
Source: Molecules. 2017 Feb 14;22(2):289. doi: 10.3390/molecules22020289 (PMC6155573; doi:10.3390/molecules22020289)
Supplement: Supplementary file 1 [file molecules-22-00289-s001.pdf]

Molecules **2017**, *22*, 289; doi:10.3390/molecules22020289

Supplementary Material: Anti-Hyperglycemic Activity of Major Compounds from *Calea ternifolia*

Sonia Escandón-Rivera, Araceli Pérez-Vásquez, Andrés Navarrete, Mariana Hernández, Edelmira Linares, Robert Bye and Rachel Mata

Table S1. Crystal data and structure refinement for Calein C.

Identification Code	172XYZ13 (Solved by: R.A. Toscano)
Empirical formula	C21 H26 O8
Formula weight	406.42
Temperature	298(2) K
Wavelength	0.71073 Å
Crystal system	Orthorhombic
Space group	P 21 21 21
Unit cell dimensions	a = 10.1880(3) Å a = 90°
	b = 12.3924(4) Å b = 90°
	c = 17.8199(6) Å g = 90°
Volume	2249.83(12) Å3
Z	4
Density (calculated)	1.200 Mg/m^3
Absorption coefficient	0.092 mm^{-1}
F (000)	864
Crystal size/colour/shape	0.316 × 0.216 × 0.212 mm/colourless/block
**Theta Range for Data Collection 2.00 to 28.28°**	
Index ranges	−13 £ h £ 12, −16 £ k £ 16, −22 £ l £ 23
Reflections collected	22646
Independent reflections	5585 [R (int) = 0.0524]
Completeness to theta	28.28° 100.0%
Measurement device	Bruker Smart Apex CCD diffractometer 01-670-01
Absorption correction	Semi-empirical from equivalents
Max. and min. transmission	0.7457 and 0.6676
Refinement method	Full-matrix least-squares on F2
Data/restraints/parameters	5585/1045/535
Goodness-of-fit on F2	1.035
Final R indices	[I >2s (I)] R 1 = 0.0670, wR 2 = 0.1847
R indices (all data)	R 1 = 0.1336, wR 2 = 0.2349
**Absolute Structure Parameter?**	
Largest diff. peak and hole	0.236 and −0.284 e.Å-3

Table S2. Atomic coordinates (×10[4]) and equivalent isotropic displacement parameters (Å^2 × 10^3) for calein C (**5**). U (eq) is defined as one third of the trace of the orthogonalized U_{ij} tensor.

	x	y	z	U(eq)
O(1)	5732(5)	5938(4)	5448(3)	83(1)
O(2)	3793(5)	2461(4)	7094(3)	75(1)
O(3)	1842(5)	4142(5)	6939(3)	61(1)
O(4)	1218(5)	5735(4)	6000(4)	92(2)
O(5)	3640(8)	6914(6)	5986(4)	90(2)
O(6)	3814(6)	2444(5)	8308(3)	107(2)
O(7)	1310(8)	5132(6)	7968(3)	83(2)
O(8)	164(9)	4379(7)	5346(6)	154(4)
C(1)	4694(7)	5518(5)	5268(3)	67(1)
C(2)	4627(9)	4513(5)	4802(4)	79(1)
C(3)	5340(8)	3622(6)	4982(4)	80(2)
C(4)	6233(6)	3477(5)	5630(4)	74(1)
C(5)	5587(6)	2801(5)	6235(4)	75(1)
C(6)	4221(6)	3178(5)	3178(5)	63(1)
C(7)	4167(5)	4328(4)	6813(3)	56(1)
C(8)	2854(5)	4901(5)	6703(3)	56(1)
C(9)	2475(5)	5229(5)	5912(3)	65(1)
C(10)	3408(7)	6037(5)	5502(3)	71(1)
C(11)	4395(6)	4068(5)	7632(3)	69(1)
C(12)	3917(6)	2949(5)	7769(3)	80(2)
C(13)	4831(9)	4684(7)	8164(4)	97(3)
C(14)	2735(9)	6464(6)	4778(4)	105(2)
C(15)	7531(7)	2927(7)	5375(6)	110(2)
C(16)	1125(6)	4381(6)	7564(3)	82(2)
C(17)	59(9)	3563(8)	7670(6)	109(2)
C(18)	−802(11)	3687(13)	8268(8)	159(5)
C(19)	−40(15)	2681(10)	7176(8)	157(4)
C(20)	123(7)	5156(7)	5742(4)	128(2)
C(21)	−1144(8)	5669(10)	6049(8)	170(4)
O(1B)	5165(11)	6319(10)	5229(6)	95(3)
O(2B)	4037(11)	2453(8)	2453(8)	78(2)
O(3B)	2038(12)	4097(10)	7091(7)	72(2)
O(4B)	959(8)	5523(9)	6169(6)	88(2)
O(5B)	3389(14)	6966(11)	6210(8)	89(4)
O(6B)	4487(16)	2159(9)	7950(7)	123(4)
O(7B)	1037(18)	5392(12)	7769(9)	103(4)
O(8B)	503(13)	4576(13)	5070(8)	102(3)
C(1B)	4099(12)	5881(9)	5165(6)	74(2)
C(2B)	3873(13)	4932(9)	4932(9)	81(2)
C(3B)	4662(14)	4044(10)	4696(7)	85(2)
C(4B)	5810(11)	3837(12)	5172(7)	87(2)
C(5B)	5481(12)	3058(10)	5804(7)	87(2)
C(6B)	4235(11)	3313(9)	6237(5)	69(2)
C(7B)	4261(9)	4370(7)	6697(5)	60(2)
C(8B)	2902(9)	4909(9)	6756(6)	58(2)
C(9B)	2301(8)	5262(9)	6035(6)	69(2)
C(10B)	2957(11)	6265(8)	5644(6)	72(2)
C(11B)	4693(12)	3961(8)	7447(6)	76(2)
C(12B)	4348(12)	2795(8)	7497(6)	80(2)
C(13B)	5282(16)	4473(11)	7989(8)	84(4)
C(14B)	1952(15)	6837(12)	5139(9)	106(4)
C(15B)	6988(13)	3391(15)	4719(9)	124(4)
C(16B)	1052(11)	4492(12)	7529(6)	86(2)
C(17B)	−1(14)	3687(16)	7673(10)	106(3)

C(18B)	−1073(17)	4020(20)	8109(13)	121(5)
C(19B)	90(20)	2633(16)	7393(14)	128(5)
C(20B)	176(10)	5231(10)	5514(7)	106(3)
C(21B)	−1066(13)	5893(15)	5520(12)	126(5)

Table S3. Bond lengths [Å] and angles [°] for calein C (**5**).

**O(1)-C(1)**	**1.223(7)**	**O(1B) -C(1B)**	**1.220(10)**
O(2)-C(12)	1.352(7)	O(2B) -C(12B)	1.362(9)
O(2) -C(6)	1.480(6)	O(2B) -C(6B)	1.469(9)
O(3) -C(16)	1.364(6)	O(3B) -C(16B)	1.363(8)
O(3) -C(8)	1.458(5)	O(3B) -C(8B)	1.464(8)
O(4) -C(20)	1.404(8)	O(4B) -C(9B)	1.425(9)
O(4) -C(9)	1.435(6)	O(4B) -C(20B)	1.460(10)
O(5) -C(10)	1.406(7)	O(5B) -C(10B)	1.402(9)
O(6) -C(12)	1.152(6)	O(6B) -C(12B)	1.137(8)
O(7) -C(16)	1.191(6)	O(7B) -C(16B)	1.194(8)
O(8) -C(20)	1.195(8)	O(8B) -C(20B)	1.182(10)
C(1) -C(2)	1.498(8)	C(1B) -C(2B)	1.495(10)
C(1) -C(10)	1.518(9)	C(1B) -C(10B)	1.519(11)
C(2) -C(3)	1.360(12)	C(2B) -C(3B)	1.364(14)
C(3) -C(4)	1.481(11)	C(3B) -C(4B)	1.467(14)
C(4) -C(5)	1.516(9)	C(4B) -C(5B)	1.520(12)
C(4) -C(15)	1.556(8)	C(4B) -C(15B)	1.548(11)
C(5) -C(6)	1.530(7)	C(5B) -C(6B)	1.519(10)
C(6) -C(7)	1.547(6)	C(6B) -C(7B)	1.546(8)
C(7) -C(11)	1.512(6)	C(7B) -C(11B)	1.496(8)
C(7) -C(8)	1.527(5)	C(7B) -C(8B)	1.541(8)
C(8) -C(9)	1.517(6)	C(8B) -C(9B)	1.488(8)
C(9) -C(10)	1.562(7)	C(9B) -C(10B)	1.573(9)
C(10) -C(14)	1.555(8)	C(10B) -C(14B)	1.536(11)
C(11) -C(13)	1.296(7)	C(11B) -C(13B)	1.302(9)
C(11) -C(12)	1.489(8)	C(11B) -C(12B)	1.490(9)
C(16) -C(17)	1.498(8)	C(16B) -C(17B)	1.488(9)
C(17) -C(18)	1.389(9)	C(17B) -C(19B)	1.401(11)
C(17) -C(19)	1.407(9)	C(17B) -C(18B)	1.404(11)
C(20) -C(21)	1.539(10)	C(20B) -C(21B)	1.508(11)
C(12) -O(2)- C(6)	111.4(4)	C(10) -O(5)- H(5)	127(5)
C(16) -O(3) -C(8)	118.3(4)	O(1) -C(1) -C(2)	122.6(7)
C(20) -O(4) -C(9)	116.7(5)	O(1) -C(1) -C(10)	119.6(5)
C(2) -C(1) -C(10)	117.8(6)	C(19) -C(17) -C(16)	119.9(6)
C(3) -C(2)- C(1)	121.3(7)	O(8) -C(20) -O(4)	125.3(7)
C(2) –C(3) -C(4)	127.6(6)	O(8) -C(20) -C(21)	124.9(7)
C(3) -C(4) -C(5)	110.8(5)	O(4) -C(20) -C(21)	109.8(6)
C(3) -C(4) -C(15)	110.3(6)	C(12B) -O(2B) -C(6B)	111.3(6)
C(5) -C(4) -C(15)	109.5(6)	C(16B) -O(3B) -C(8B)	115.4(8)
C(4) -C(5) -C(6)	115.2(5)	C(9B) -O(4B) -C(20B)	109.5(7)
O(2) -C(6) -C(5)	107.1(5)	C(10B) -O(5B) -H(5)	114(4)
O(2) -C(6) -C(7)	104.8(4)	O(1B) -C(1B) -C(2B)	122.9(9)
C(5) -C(6) -C(7)	115.0(4)	O(1B) -C(1B) -C(10B)	119.4(8)
C(11) -C(7) -C(8)	110.9(4)	C(2B) -C(1B) -C(10B)	117.6(8)
C(11) -C(7) -C(6)	99.9(4)	C(3B) -C(2B) -C(1B)	121.2(10)
C(8) -C(7) -C(6)	114.2(4)	C(2B) -C(3B) -C(4B)	129.5(10)
O(3) -C(8) -C(9)	105.1(4)	C(3B) -C(4B) -C(5B)	111.3(9)
O(3) -C(8) -C(7)	106.4(3)	C(3B) -C(4B) -C(15B)	112.3(10)
C(9) -C(8) -C(7)	117.8(4)	C(5B) -C(4B) -C(15B)	109.3(9)
O(4) -C(9) -C(8)	104.0(4)	C(6B) -C(5B) -C(4B)	115.4(8)
O(4) -C(9) -C(10)	108.3(4)	O(2B) -C(6B) -C(5B)	107.9(8)

C(8) -C(9) -C(10)	116.8(4)	O(2B) -C(6B) -C(7B)	105.1(5)
O(5) -C(10) -C(1)	110.5(5)	C(5B) -C(6B) -C(7B)	115.5(7)
O(5) -C(10) -C(14)	108.7(5)	C(11B) -C(7B) -C(8B)	110.5(7)
C(1) -C(10) -C(14)	107.2(6)	C(11B) -C(7B) -C(6B)	101.0(5)
O(5) -C(10) -C(9)	108.2(5)	C(8B) -C(7B) -C(6B)	112.8(6)
C(1) -C(10) -C(9)	112.4(5)	O(3B) -C(8B) -C(9B)	107.9(7)
C(14) -C(10) -C(9)	109.7(5)	O(3B) -C(8B) -C(7B)	105.7(6)
C(13) -C(11) -C(12)	122.7(5)	C(9B) -C(8B) -C(7B)	116.0(7)
C(13) -C(11) -C(7)	129.3(5)	O(4B) -C(9B) -C(8B)	108.5(7)
C(12) -C(11) -C(7)	107.9(4)	O(4B) -C(9B) -C(10B)	107.6(7)
O(6) -C(12) -O(2)	119.4(6)	C(8B) -C(9B) -C(10B)	116.1(7)
O(6) -C(12) -C(11)	132.3(6)	O(5B) -C(10B) -C(1B)	110.9(7)
O(2) -C(12) -C(11)	107.5(4)	O(5B) -C(10B) -C(14B)	110.1(8)
O(7) -C(16) -O(3)	125.4(5)	C(1B) -C(10B) -C(14B)	109.0(8)
O(7) -C(16) -C(17)	124.5(5)	O(5B) -C(10B) -C(9B)	107.7(7)
O(3) -C(16) -C(17)	110.1(5)	C(1B) -C(10B) -C(9B)	109.1(7)
C(18) -C(17) -C(19)	121.4(7)	C(14B) -C(10B) -C(9B)	109.9(7)
C(18) -C(17) -C(16)	118.7(7)	C(13B) -C(11B)-C(12B)	122.5(8)
C(13B) -C(11B) -C(7B)	129.3(8)	O(3B) -C(16B) -C(17B)	112.9(7)
C(12B) -C(11B) -C(7B)	108.2(5)	C(19B)- C(17B)-C(18B)	121.8(9)
O(6B) -C(12B) -O(2B)	117.8(9)	C(19B) -C(17B)-C(16B)	121.1(8)
O(6B) -C(12B) -C(11B)	133.3(9)	C(18B) -C(17B)-C(16B)	117.1(8)
O(2B) -C(12B) -C(11B)	107.6(6)	O(8B) -C(20B)- O(4B)	123.5(8)
O(7B) -C(16B) -O(3B)	123.4(8)	O(8B) -C(20B) -C(21B)	128.0(10)
O(7B) -C(16B) -C(17B)	123.8(8)	O(4B) -C(20B)-C(21B)	108.5(8)

Table S4. Hydrogen coordinates (×10[4]) and isotropic displacement parameters (Å^2 × 10^3) for calein C (**5**).

	x	y	z	(eq)
H(5)	4320(70)	7040(50)	6240(30)	135
H(2)	4088	4499	4381	95
H(3)	5258	3035	4660	96
H(4)	6442	4188	5839	89
H(5A)	6154	2796	6672	90
H(5B)	5521	2064	6056	90
H(6)	3609	3122	6054	76
H(7)	4887	4770	6616	67
H(8)	2823	5538	7028	68
H(9)	2375	4579	5603	78
H(13A)	4855	4432	8655	117
H(13B)	5120	5378	8055	117
H(14A)	3373	6822	4470	157
H(14B)	2053	6963	4911	157
H(14C)	2363	5869	4505	157
H(15A)	8088	2816	5802	165
H(15B)	7971	3380	5018	165
H(15C)	7335	2244	5147	165
H(18A)	−1461	3181	8348	8348
H(18B)	−720	4277	8587	191
H(19A)	−754	2226	7329	235
H(19B)	764	2276	7186	235
H(19C)	−196	2939	6676	235
H(21A)	−1776	5731	5652	254
H(21B)	−951	6373	6245	254
H(21C)	−1495	5224	6441	254
H(2B)	3181	4948	4323	98
H(3B)	4439	3488	4369	102
H(4B)	6078	4523	5398	105
H(5C)	5401	2339	5594	104
H(5D)	6211	3048	6154	104
H(6B)	3488	3327	5890	83
H(7B)	4908	4875	6491	72
H(8B)	2962	5530	7095	70
H(9B)	2331	4654	5684	82

H(13C)	5503	4110	8428	101
H(13D)	5481	5202	7938	101
H(14D)	2371	7424	4882	159
H(14E)	1247	7111	5441	159
H(14F)	1611	6334	4779	159
H(15D)	7428	3975	4470	186
H(15E)	6677	2884	4352	186
H(15F)	7588	3037	5053	186
H(18C)	−1747	3545	8219	145
H(18D)	−1109	4729	8288	145
H(19D)	−672	2230	7541	192
H(19E)	863	2292	7591	192
H(19F)	143	2655	6855	192
H(21D)	−1673	5591	5874	189
H(21E)	−1451	5888	5028	189
H(21F)	−863	6622	5661	189

Table S5. Hydrogen bonds for calein C (**5**).

D-H...A	d(D-H)	d(H...A)	d(D...A)	<(DHA)
O(5)-H(5)...O(6)#1	0.84(6)	2.12(7)	2.956(9)	170(6)

Symmetry transformations used to generate equivalent atoms: #1 –x + 1, y + 1/2,−z + 3/2.

Table S6. Effect of calein A (**4**) on blood glucose levels in normoglycemic and NA/STZ mice during an OSTT [a].

	**Blood Glucose Concentration (mg/dL)**					
Test Samples (mg/Kg of BW)	0 h	0.5 h	1 h	1.5 h	2 h	3 h
			**Normal Mice**			
Vehicle	121 ± 2	200 ± 7	137 ± 8	140 ± 8	124 ± 6	106 ± 9
Acarbose (5)	124 ± 10	148 ± 5 *	156 ± 4	136 ± 10	119 ± 5	120 ± 9
1 (3.16)	122 ± 5	152 ± 5 *	153 ± 7	154 ± 8	160 ± 7	127 ± 6
2 (7)	123 ± 4	144 ± 6 *	163 ± 8	164 ± 6	127 ± 5	117 ± 3
3 (10)	120 ± 12	133 ± 17 *	130 ± 22	130 ± 14	134 ± 15	113 ± 11
			**NA/STZ Mice**			
Vehicle	192 ± 6	371 ± 10	312 ± 11	287 ± 12	258 ± 12	213 ± 11
Acarbose (5)	197 ± 10	225 ± 11 *	221 ± 13 *	210 ± 14 *	205 ± 12 *	187 ± 14
1 (3.16)	215 ± 15	250 ± 19 *	223 ± 23 *	220 ± 21 *	223 ± 29	188 ± 18
2 (7)	189 ± 21	189 ± 13 *	187 ± 13 *	197 ± 18 *	202 ± 23	178 ± 12
3 (10)	202 ± 22	262 ± 25 *	234 ± 26 *	231 ± 24 *	229 ± 22	217 ± 18

[a] Each value is the mean ± SEM for six mice in each group. * $p < 0.05$ significantly different ANOVA followed by Dunnett's t test for comparison with respect to control group.

Table S7. Effect of calein C (**5**) on blood glucose levels in normoglycemic and NA/STZ mice during an OSTT [a].

	**Blood Glucose Concentration (mg/dL)**					
Test Samples (mg/Kg of BW)	0 h	0.5 h	1 h	1.5 h	2 h	3 h
			**Normal Mice**			
Vehicle	121 ± 2	200 ± 7	137 ± 8	140 ± 8	124 ± 6	106 ± 9
Acarbose (5)	124 ± 8	148 ± 5 *	156 ± 4	136 ± 10	119 ± 5	120 ± 9
1 (3.16)	129 ± 2	169 ± 4 *	148 ± 7	146 ± 7	133 ± 7	125 ± 4
2 (7)	125 ± 5	171 ± 8 *	160 ± 3	158 ± 4	144 ± 5	129 ± 5
3 (10)	118 ± 9	149 ± 10 *	157 ± 7	139 ± 9	124 ± 5	120 ± 4
			**NA/STZ Mice**			
Vehicle	218 ± 24	355 ± 14	292 ± 15	274 ± 13	250 ± 10	215 ± 12
Acarbose (5)	197 ± 10	225 ± 11 *	221 ± 13 *	210 ± 14 *	205 ± 12	187 ± 14
1 (3.16)	195 ± 5	289 ± 16 *	238 ± 18 *	223 ± 10 *	223 ± 15	186 ± 12
2 (7)	229 ± 18	246 ± 16 *	211 ± 17 *	187 ± 8 *	193 ± 13 *	192 ± 10

3 (10)	199 ± 12	276 ± 20 *	249 ± 14	237 ± 12	251 ± 24	243 ± 20

[a] Each value is the mean ± SEM for six mice in each group. * $p < 0.05$ significantly different ANOVA followed by Dunnett's t test for comparison with respect to control group.

Table S8. Effect of chromene **1** on blood glucose levels in normoglycemic and NA/STZ mice during an OSTT [a].

	Blood Glucose Concentration (mg/dL)					
Test Samples (mg/Kg of BW)	0 h	0.5 h	1 h	1.5 h	2 h	3 h
**Normal Mice**						
Vehicle	121 ± 2	200 ± 7	137 ± 8	140 ± 8	124 ± 6	106 ± 9
Acarbose (5)	124 ± 8	148 ± 5 *	156 ± 4	136 ± 10	119 ± 5	120 ± 9
1 (5.6)	113 ± 1	165 ± 10 *	120 ± 7	115 ± 5	133 ± 5	99 ± 5
2 (10)	125 ± 2	163 ± 9 *	123 ± 5	120 ± 9	125 ± 8	117 ± 9
3 (31.6)	123 ± 3	176 ± 6 *	135 ± 7	131 ± 7	119 ± 7	112 ± 7
**NA/STZ Mice**						
Vehicle	192 ± 6	371 ± 10	312 ± 11	287 ± 12	258 ± 12	213 ± 11
Acarbose (5)	197 ± 10	225 ± 11 *	221 ± 13 *	210 ± 14 *	205 ± 12 *	187 ± 14
1 (5.6)	199 ± 18	290 ± 13 *	248 ± 11 *	247 ± 9	246 ± 9	225 ± 11
2 (10)	190 ± 18	259 ± 18 *	247 ± 14 *	245 ± 16	256 ± 18	226 ± 12
3 (31.6)	174 ± 10	240 ± 16 *	241 ± 12 *	245 ± 13	248 ± 18	215 ± 16

[a] Each value is the mean ± SEM for six mice in each group. * $p < 0.05$ significantly different ANOVA followed by Dunnett's t test for comparison with respect to control group.

Table S9. Effect of essential oil of *C. ternifolia* on blood glucose levels in normoglycemic and NA/STZ mice during an OSTT [a].

	Blood Glucose Concentration (mg/dL)					
Test Samples (mg/Kg of BW)	0 h	0.5 h	1 h	1.5 h	2 h	3 h
**Normal Mice**						
Vehicle	127 ± 6	200 ± 7	146 ± 9	150 ± 11	140 ± 11	118 ± 10
Acarbose (5)	141 ± 8	169 ± 7 *	149 ± 7	153 ± 5	142 ± 5	118 ± 6
1 (31.6)	137 ± 5	154 ± 6 *	160 ± 3	145 ± 5	130 ± 6	118 ± 5
2 (100)	137 ± 5	159 ± 10 *	151 ± 8	136 ± 9	137 ± 10	115 ± 7
3 (316)	141 ± 7	153 ± 7 *	162 ± 7	147 ± 5	133 ± 5	115 ± 6
**NA/STZ Mice**						
Vehicle	244 ± 19	428 ± 14	346 ± 25	301 ± 28	269 ± 27	268 ± 28
Acarbose (5)	238 ± 38	256 ± 30 *	229 ± 21 *	214 ± 23 *	174 ± 27 *	153 ± 20 *
1 (31.6)	209 ± 18	320 ± 32 *	251 ± 33 *	224 ± 33	179 ± 25 *	173 ± 22 *
2 (100)	212 ± 30	299 ± 27 *	244 ± 30 *	216 ± 25 *	186 ± 22 *	178 ± 19 *
3 (316)	221 ± 30	314 ± 24 *	253 ± 31 *	241 ± 28	190 ± 25 *	172 ± 17 *

[a] Each value is the mean ± SEM for six mice in each group. * $p < 0.05$ significantly different ANOVA followed by Dunnett's t test for comparison with respect to control group.

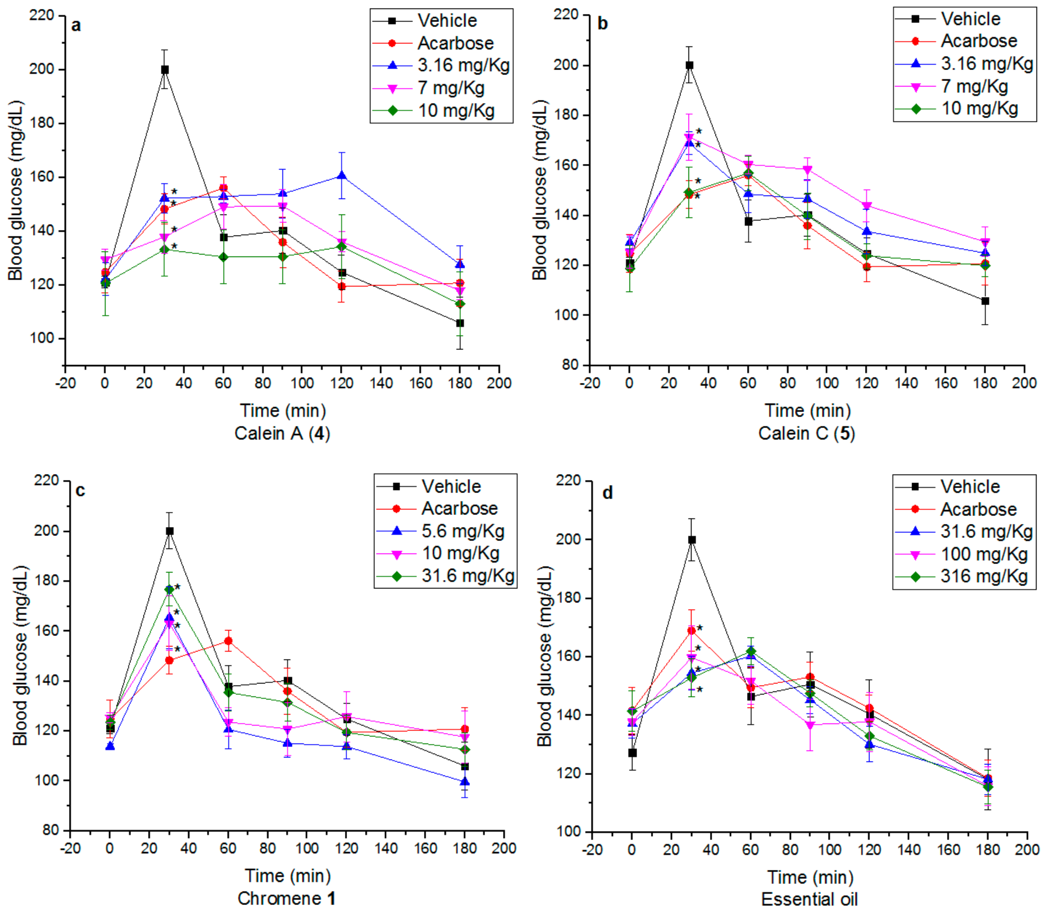

Figure S1. Effect of (**a**) calein A (**4**); (**b**) calein C (**5**) (**c**) chromene **1** and (**d**) Essential oil in normoglycemic mice, after a normal sucrose load (3 g/kg). * $p < 0.05$ significantly different ANOVA followed by Dunnett´s *t* test for comparision with respect to vehicle.

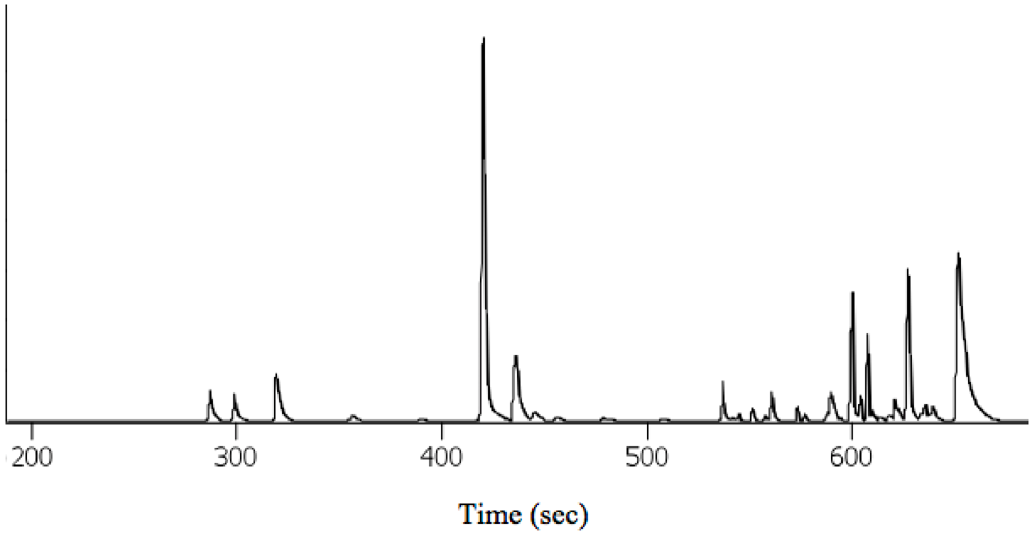

Figure S2. Total ion current chromatogram of the essential oil from *C. ternifolia.* For chromatographic (GC-MS) conditions, see the Experimental Section.

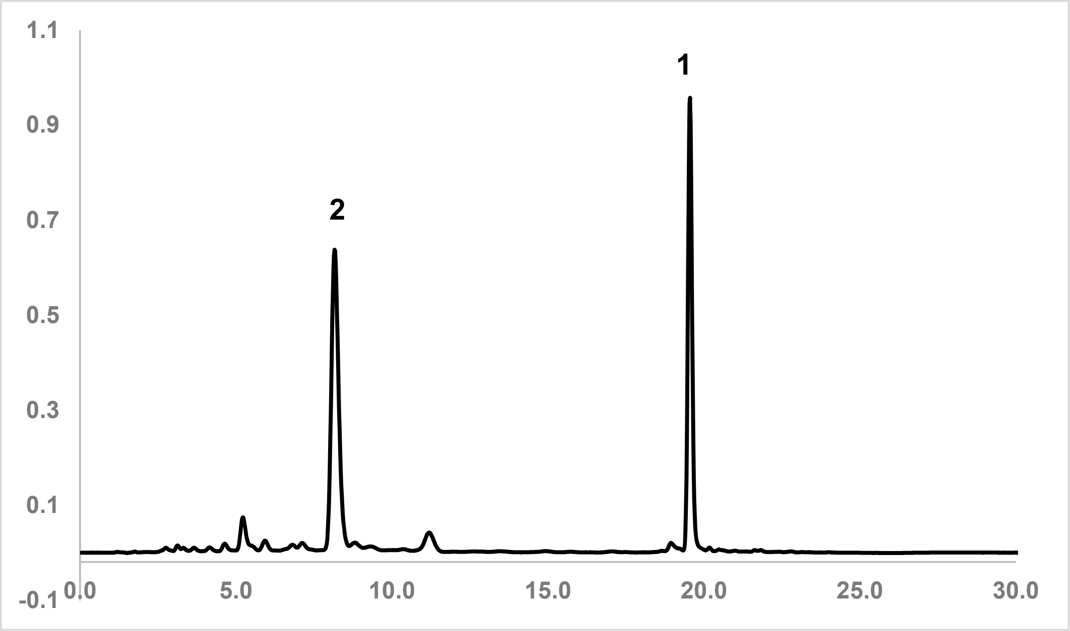

Figure S3. HPLC-DAD chromatogram of the chromene-rich fraction (CRF) from *C. ternifolia* aqueous extract; detection wavelength 265 nm. For chromatographic conditions, see the Experimental Section.

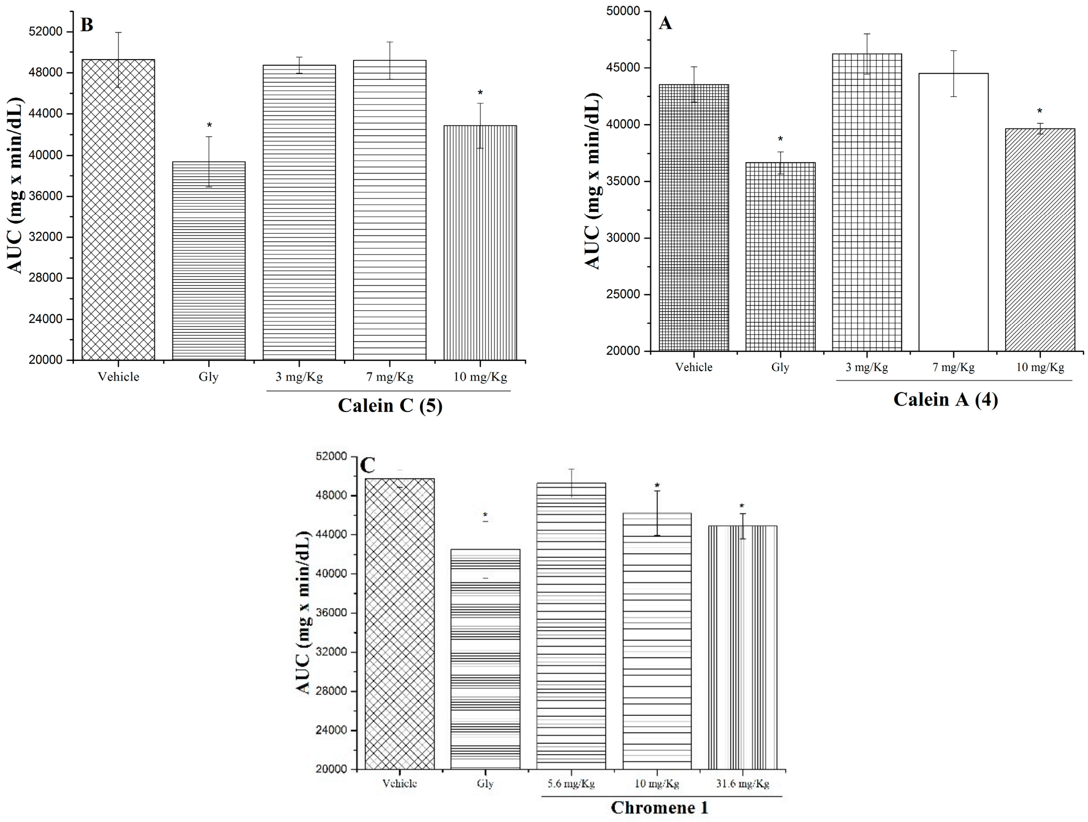

Figure S4. Hypoglycemic action of A) calein A (**4**), B) calein C (**5**) and C) chromene **1** in normoglycemic mice. AUC: area under the curve, Gly: glibenclamide. Each bar represents the mean ± SEM for 6 mice in each group. * $p < 0.05$, significantly different ANOVA followed by Dunnett *post hoc* test for comparison with respect to vehicle control.

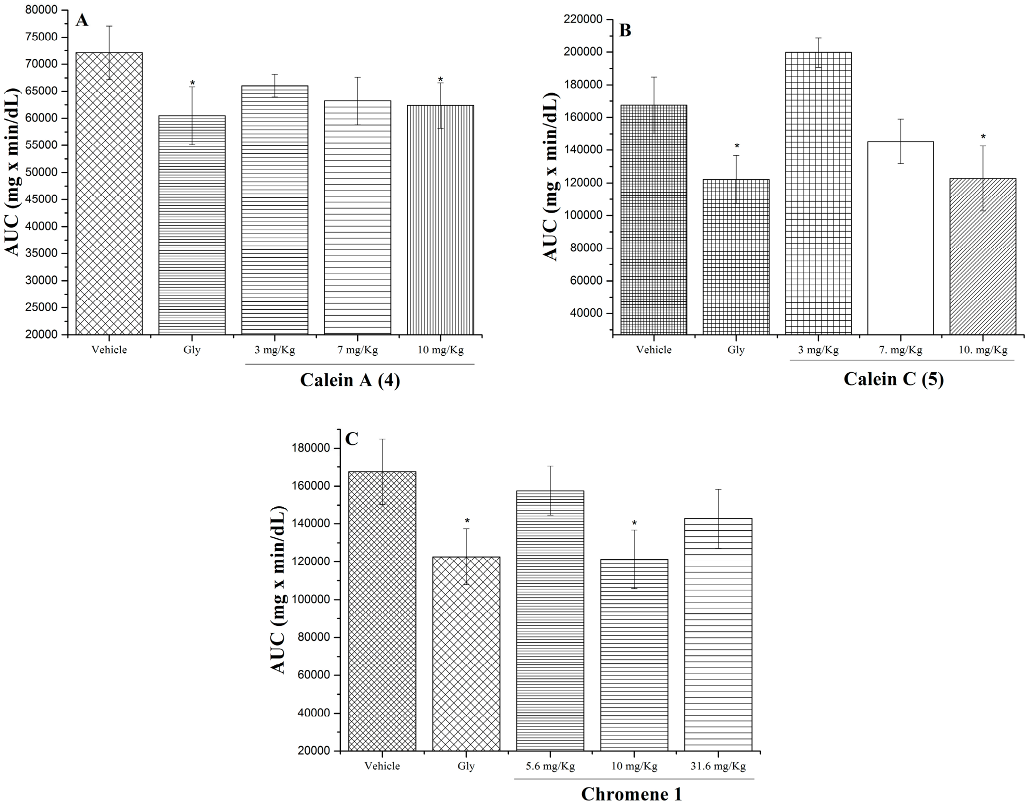

Figure S5. Hypoglycemic action of A) calein A (**4**), B) calein C (**5**) and C) chromene **1** in NA-STZ-treated mice. AUC: area under the curve [(mg/dL) x min], Gly: glybenclamide. Each bar represents the mean ± SEM for 6 mice in each group. *$p < 0.05$, significantly different ANOVA followed by Dunnett *post hoc* test for comparison with respect to vehicle control.
